# Genetic association and bidirectional Mendelian randomization for causality between gut microbiota and six lung diseases

**DOI:** 10.3389/fmed.2023.1279239

**Published:** 2023-12-15

**Authors:** Yue Su, Youqian Zhang, Jinfu Xu

**Affiliations:** ^1^Department of Respiratory and Critical Care Medicine, School of Medicine, Shanghai Pulmonary Hospital, Tongji University, Shanghai, China; ^2^Health Science Center, Yangtze University, Hubei Province, Jingzhou, China

**Keywords:** Mendelian randomization, gut microbiota, lung diseases, lung function, chronic lung diseases

## Abstract

**Purposes:**

Increasing evidence suggests that intestinal microbiota correlates with the pathological processes of many lung diseases. This study aimed to investigate the causality of gut microbiota and lung diseases.

**Methods:**

Genetic information on intestinal flora and lung diseases [asthma, chronic bronchitis, chronic obstructive pulmonary disease (COPD), interstitial lung disease (ILD), lower respiratory tract infection (LRTI), pulmonary arterial hypertension (PAH)] and lung function was obtained from UK Biobank, FinnGen, and additional studies. A Mendelian randomization (MR) analysis was conducted to explore the causal association between gut microbiota and lung diseases.

**Results:**

The genetic liability to lung diseases may be associated with the abundance of certain microbiota taxa. Specifically, the genus *Prevotella* (*p* = 0.041) was related to a higher risk of asthma; the family Defluviitaleaceae (*p* = 0.002) and its child taxon were identified as a risk factor for chronic bronchitis; the abundance of the genus *Prevotella* (*p* = 0.020) was related to a higher risk of ILD; the family Coriobacteriaceae (*p* = 0.011) was identified to have a positive effect on the risk of LRTI; the genus *Lactobacillus* (*p* = 0.0297) has been identified to be associated with an increased risk of PAH, whereas the genus *Holdemanella* (*p* = 0.0154) presented a causal decrease in COPD risk; the order Selenomonadales was identified to have a positive effect on the risk of FEV1(*p* = 0.011). The reverse TSMR analysis also provided genetic evidence of reverse causality from lung diseases to the gut microbiota.

**Conclusion:**

This data-driven MR analysis revealed that gut microbiota was causally associated with lung diseases, providing genetic evidence for further mechanistic and clinical studies to understand the crosstalk between gut microbiota and lung diseases.

## Introduction

Respiratory diseases are the leading causes of disability and death worldwide (1) because the lung is a complex and vulnerable organ that is exposed to smoking, environmental degradation, and occupational hazards (2). According to the systematic analysis for the Global Burden Disease Study 2019, lower respiratory infection is the 3rd cause of death. Chronic obstructive pulmonary disease (COPD) is the 6th cause of death (3), and more than 500 million people have chronic respiratory diseases across the world (1). Regardless of the pathophysiological process of infectious or chronic respiratory diseases, the overwhelming immune responses and improper reparative and regenerative processes account for lung structural and functional disorders (4). Gut microbiota is the community of microorganisms living in the digestive tracts, playing a vital role in training host immunity, modulating endocrine function and metabolic rewiring, and producing various biological compounds that affect the host (5). However, the composition of the human gut microbiome is determined and dynamically altered by genetic or exogenous factors, such as diseases, diets, and aging (6). It has been demonstrated that different respiratory diseases can be affected by changes in the intestinal microenvironment and vice versa (7). Emerging studies have indicated that gut microbial species and their derived functional metabolites regulate lung homeostasis, and the dysbiosis of the gut-lung axis contributes to the development and progression of respiratory diseases (8), suggesting that gut microbiota may be a potential causal factor of respiratory diseases. Moreover, gut microbiome-derived small-chain fatty acids (SCFAs) are capable of activating bone marrow hematopoiesis (9). Chiu et al. have shown that the mean proportions of *Acinetobacter* and *Stenotrophomonas* are significantly elevated in COPD patients. Similarly, Wang et al. have found that gut microbiota-derived succinate aggravates acute lung injury after ischemia/reperfusion in mice (10).

However, due to the lack of evidence from randomized controlled studies, it remains unclear whether there is a causality between gut microbiota and lung diseases and lung function. Previous family-based or population-based studies have suggested that many respiratory diseases are associated with genetic variation, and genome-wide association studies (GWAS) have shown that many genetic variants are related to pulmonary traits (11, 12). An MR analysis is capable of employing genetic variants as proxies of exposure to yield the causal estimate of the environmental exposure on the intended outcomes (13) using GWAS, which provides a high degree of evidence and a low susceptibility to confounding factors. Importantly, MR overcomes the constraints of conventional observational studies, such as potential bias from confounding and reverse causation, and produces reliable results (14).

Herein, this study aimed to investigate the causal relationship between gut microbiota and lung diseases and lung function. Using a reverse MR approach, we also explore whether SNPs associated with lung diseases and lung function are causally related to gut microbiota.

## Methods

### Study settings

**Figure 1:**
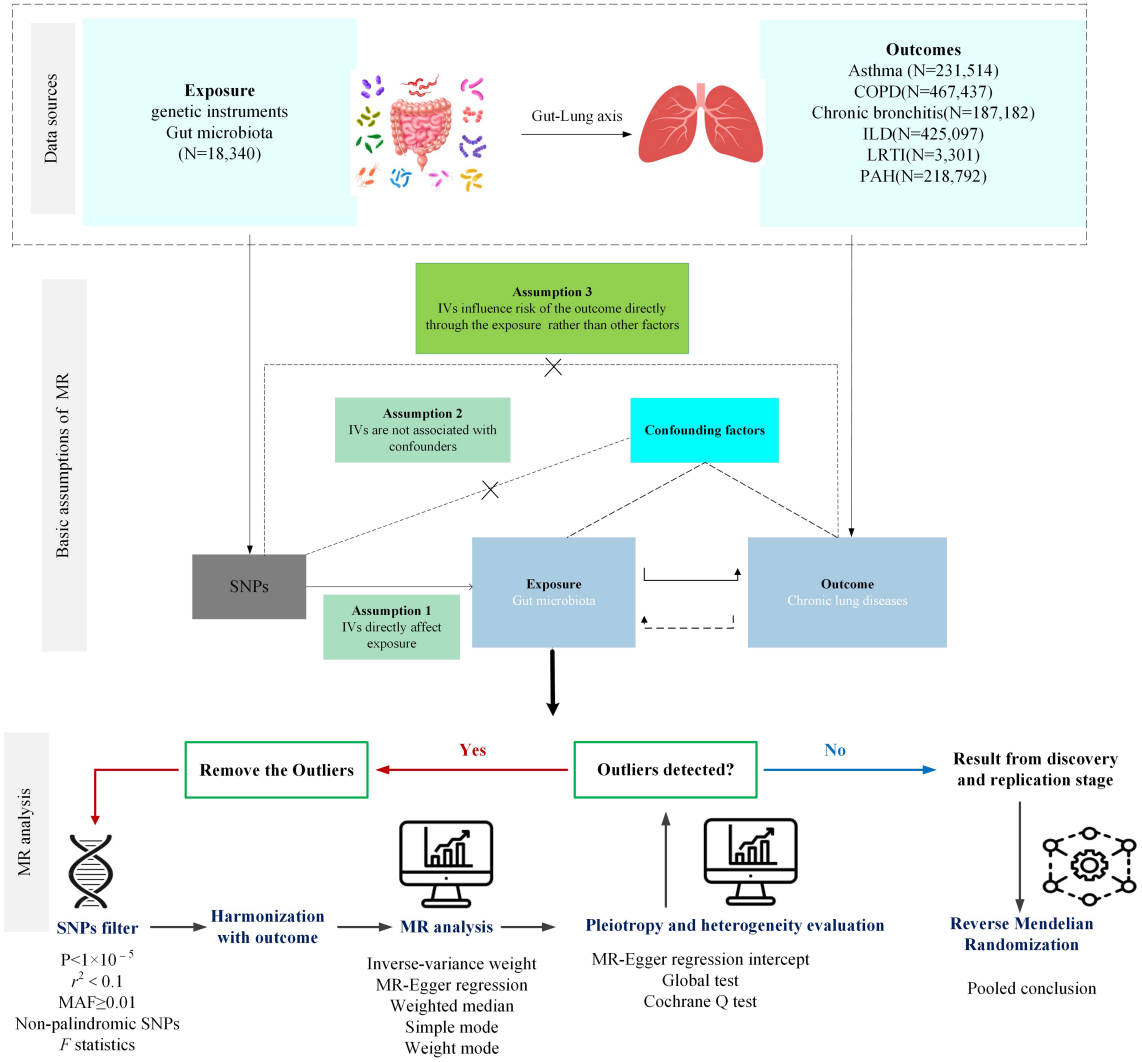
Flowchart of MR analysis and assumptions.

A bidirectional two-sample MR (TSMR) analysis was designed to assess the causal relationship between gut microbiota and the risk of lung diseases and lung function. The forward MR analysis was performed to explore the causal effect of each taxon on lung diseases and lung function, while the reverse MR was performed to investigate whether the genetic liability for lung diseases and lung function influenced the abundance of the gut microbiota. The study flowchart is presented in Figure 1.

### Data sources

#### Exposure data sources

Summary statistics of the intestinal flora (211 bacterial taxa) were obtained from a genome-wide meta-analysis by the MiBioGen consortium, comprising 18,340 participants from 24 European cohorts with 122,110 loci of variation (15). After removing 15 taxa without specific species names, 196 bacterial traits (119 genera, 32 families, 20 orders, 16 classes, and 9 phyla) were screened.

#### Outcome data sources

The traits involved in this study were downloaded in the IEU Open GWAS project (updated to 2023.05.31, *N* = 42,346) or FinnGen (https://www.finngen.fi/fi). The genetic variants included in this study were, all or partially, identified from the UK Biobank (16) or FinnGen Research.

#### Selection of instrument variants

After removing 15 taxa without specific species names, 196 bacterial traits (119 genera, 32 families, 20 orders, 16 classes, and 9 phyla) were screened. Due to the limited number of SNPs available, a locus-wide significance threshold (1 × 10^−5^) was adopted to find more potential SNPs related to the outcome, and the minor allele frequency (MAF) threshold with the IVs of interest was 0.01. To ensure the independence of the selected SNPs, the linkage disequilibrium (LD) test was conducted using LD *r*^2^ < 0.1 within a clumping distance of 500 kb. However, if SNPs could not be found in the outcome datasets, proxies at the threshold of LD *r*^2^ > 0.8 were used if applicable. To avoid weak instrument bias, the F-statistic of each SNP was calculated, and the SNP with *F* < 10 was removed (17). Finally, the process of harmonizing was performed to eliminate the SNPs with incompatible or palindromic (e.g., A/T or G/C alleles) with intermediate allele frequencies (e.g., A/C paired with A/G), and the number of SNPs included in the analysis was more than three.

### MR analysis and sensitivity analysis

TSMR was performed to analyze the causality between gut microbiota and lung diseases and lung function. The inverse-variance weighted (IVW) method was adopted as the main method to preliminarily assess the potential causal effects of each bacterial taxon on chronic lung diseases and lung function in the absence of horizontal pleiotropic effects. If the result of the IVW method was statistically significant (*p* < 0.05), a potential causal association between the bacterial taxa and disease was considered. Simultaneously, Cochrane’s Q test was used to assess the heterogeneity between IVs, and if heterogeneity was observed (*p* < 0.05), the random-effects IVW model was used to provide a more conservative estimate; otherwise, the fixed-effect IVW model would be applied. Weight median (WM) method, MR-Egger regression, simple mode, and weight mode are the other four MR methods to explore the causality and provide wider confidence intervals (18), of which the WM method could provide a consistent estimate if at least half of the weight comes from valid IVs (19); MR-Egger regression assumes that more than 50% of IVs are influenced by horizontal pleiotropy (20). Similarly, simple mode and weight mode are complementary methods to investigate the causality of the exposure and outcomes.

To test the sensitivity of the results of the above MR analysis, the MR-Egger intercept test and MR-PRESSO global test were applied to test the horizontal pleiotropy among the selected IVs. Leave-one-out analysis was conducted to detect and remove any potential outliers that affect the observed causal correlation. In terms of the significant MR estimates, the Mendelian median pleiotropy residual sum and outlier (MR-PRESSO) test were used to assess the heterogeneity. In detail, the MR-PRESSO global test was used to test whether there exists a horizontal pleiotropy, and the MR-PRESSO outlier test was calculated to remove outliers to adjust horizontal pleiotropy. The value of distributions in the MR-PRESSO analysis was set to 1,000 (21).

## Results

### Causal effects of gut microbiota and lung diseases

**Figure 2:**
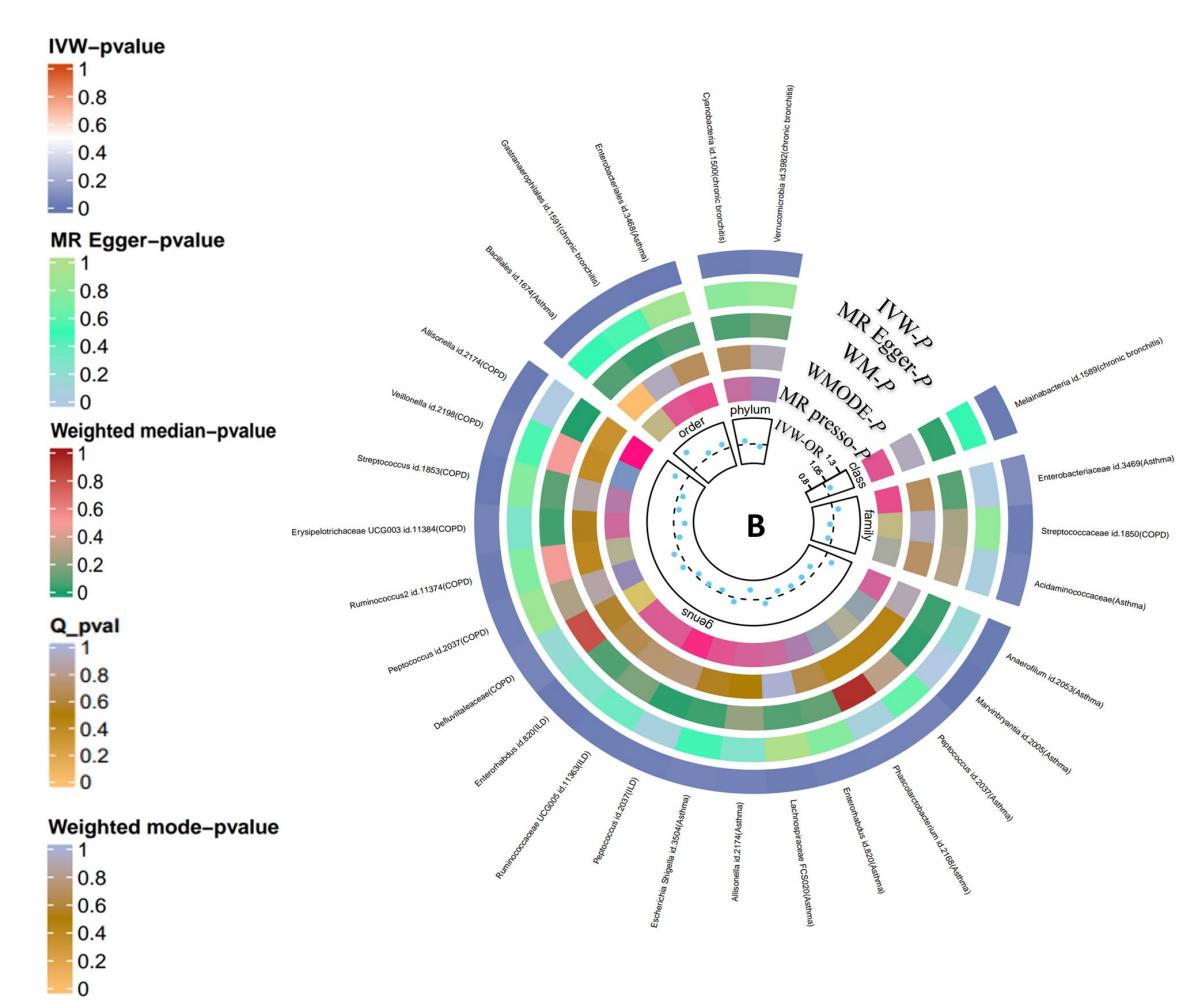
Causal analysis of gut microbiota on lung diseases (locus-wide significance, *p* < 1*10^−5^). From the inner to outer circles, they represent the estimates of: MR-PRESSO, weight mode, weight median, MR-Egger, inverse-variance weighted methods, respectively. And the shades of color reflect the magnitude of *p*-value.

**Figure 3:**
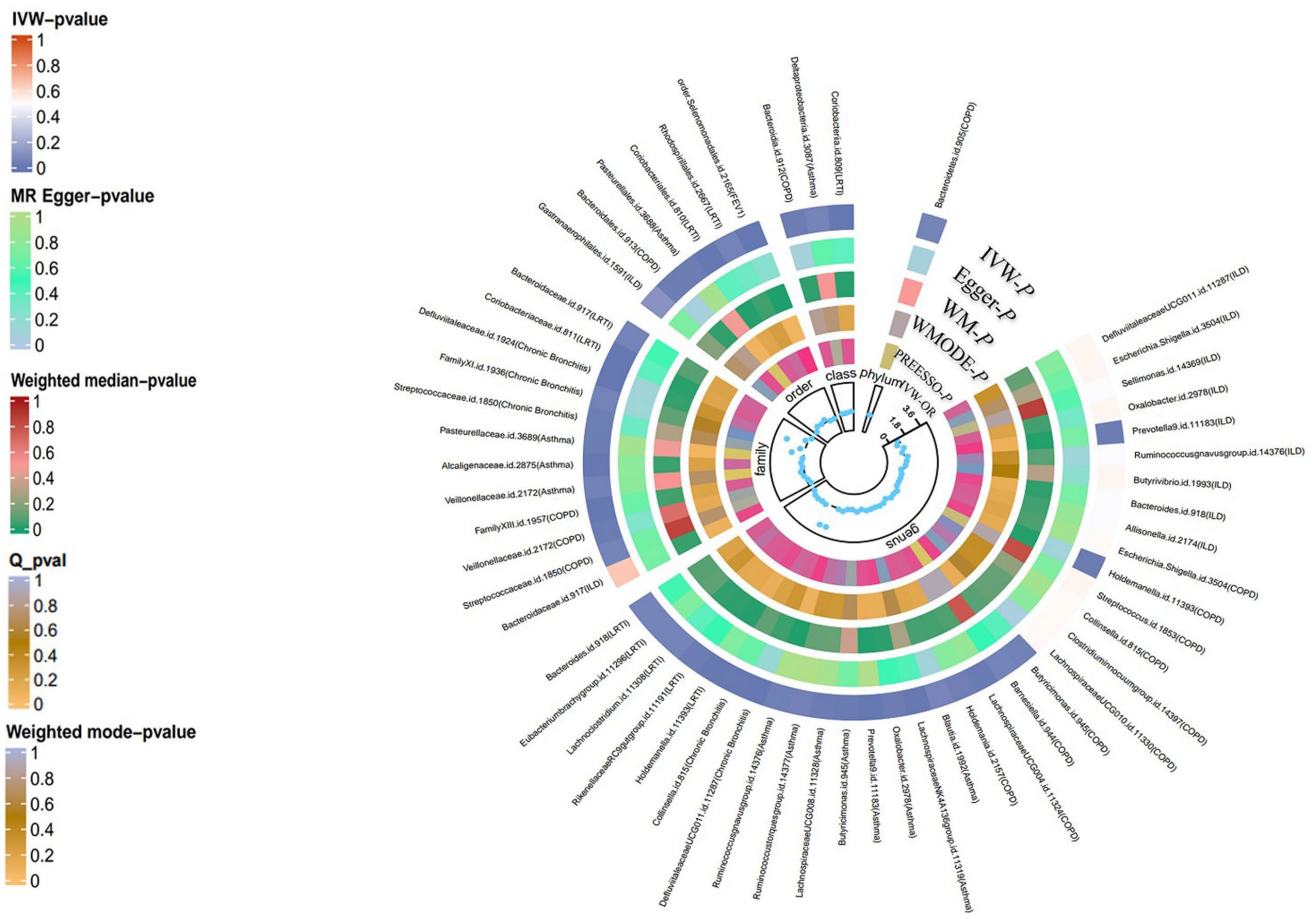
Summary-level MR analysis of lung diseases on gut microbiota (locus-wide significance, *p* < 1*10^−5^). From the inner to outer circles, they represent the estimates of: MR-PRESSO, weight mode, weight median, MR-Egger, inverse-variance weighted methods, respectively. And the shades of color reflect the magnitude of *p*-value.

According to the process of selection described above, SNPs of each respiratory disease were screened. The details of the SNPs involved in the TSMR analysis for asthma, COPD, chronic bronchitis, ILD, LRTI, PAH, and lung function are shown in Supplementary Tables S1–S7. Potential causal relationships between gut microbiota and lung diseases were found using TSMR methods before Benjamin and Hochberg correction (Figures 2, 3). MR results and sensitivity analysis of the significant relationship between gut microbiota and six lung diseases are shown in Tables 1, 2.

**Table 1:** MR results of causality of gut microbiota on lung diseases (*p* < 1 × 10^−5^).

Exposure	Outcome	Bacterial taxa	N.SNP	IVW		MR-Egger intercept			Cochrane’s Q		
				OR	*p*	Intercept	SE	*p*	*Q*	*Q_df*	*Q_p*
Gut microbiota	Asthma	Genus *Prevotella*	15	1.141	4.06E-02	0.014	0.011	0.216	10.981	14	0.688
	COPD	Genus *Holdemanella*	11	0.850	1.54E-02	−0.036	0.028	0.214	6.568	*10*	0.766
	Chronic bronchitis	Genus *Defluviitaleaceae*	9	3.264	2.21E-02	−0.079	0.125	0.548	8.883	*8*	0.352
	Chronic bronchitis	Family *Defluviitaleaceae*	11	3.087	2.17E-03	−0.067	0.099	0.515	9.607	10	0.476
	ILD	Genus *Prevotella*	15	1.347	2.05E-02	0.043	0.027	0.136	21.541	14	0.089
	LRTI	Family *Coriobacteriaceae*	13	1.289	1.06E-02	0.001	0.020	0.977	7.998	12	0.785
	PAH	Genus *Lactococcus*	9	5.593	2.97E-02	−0.102	0.187	0.603	6.001	8	0.647
	FEV1	Order *Selenomonadales*	12	0.931	6.90E-03	0.002	0.005	0.665	18.050	11	0.080

*p* means *p* value. *Q_df* means degrees of freedom.

**Table 2:** MR results of causality of lung diseases on gut microbiota (*p* < 1 × 10^−5^).

Exposure	Outcome	Bacterial taxa	N.SNP	IVW		MR-Egger intercept			Cochrane’s Q		
				OR	*p*	Intercept	SE	*p*	*Q*	*Q_df*	*Q_p*
Asthma	Gut microbiota	family *Acidaminococcaceae*	26	0.942	4.90E-02	0.020	0.012	0.118	20.812	25	0.703
Asthma		family *Enterobacteriaceae*	27	1.083	5.60E-03	0.007	0.012	0.527	22.227	26	0.676
Asthma		order *Bacillales*	19	1.271	7.94E-03	0.001	0.037	0.982	34.485	18	0.011
Asthma		order *Enterobacteriales*	27	1.083	5.59E-03	0.007	0.012	0.527	22.227	26	0.676
Asthma		genus *Allisonella*	14	0.850	3.64E-02	−0.053	0.029	0.097	12.434	13	0.492
Asthma		genus *Escherichia Shigella*	25	1.065	4.06E-02	−0.002	0.012	0.852	23.652	24	0.482
Asthma		genus *Phascolarctobacterium*	26	0.936	3.91E-02	0.017	0.013	0.198	25.246	25	0.449
Asthma		genus *Anaerofilum*	24	1.128	1.16E-02	−0.016	0.019	0.405	14.798	23	0.902
Asthma		genus *Enterorhabdus*	26	0.922	3.41E-02	−0.015	0.015	0.355	22.069	25	0.632
Asthma		genus *Lachnospiraceae*	26	1.070	1.79E-02	0.007	0.012	0.521	11.690	25	0.989
Asthma		genus *Marvinbryantia*	26	0.890	1.54E-04	0.032	0.012	0.015	25.193	25	0.452
Asthma		genus *Peptococcus*	26	0.914	4.36E-03	−0.001	0.018	0.963	25.284	25	0.447
COPD		family *Streptococcaceae*	58	0.946	1.85E-02	−0.006	0.006	0.300	50.268	57	0.724
COPD		genus *Defluviitaleaceae*	58	1.068	4.84E-02	0.017	0.008	0.039	48.007	57	0.796
COPD		genus *Peptococcus*	58	1.093	3.35E-02	0.009	0.010	0.378	61.679	57	0.312
COPD		genus *Ruminococcus*	58	0.934	2.15E-02	0.003	0.010	0.794	68.038	57	0.150
COPD		genus *Erysipelotrichaceae*	6	0.833	3.97E-02	0.036	0.037	0.396	4.054	5	0.542
		genus *Streptococcus*	58	0.934	3.80E-03	−0.004	0.006	0.523	44.913	57	0.877
COPD		genus *Veillonella*	58	0.931	2.58E-02	−0.011	0.008	0.169	59.643	57	0.380
COPD		genus *Allisonella*	46	1.169	1.03E-02	−0.028	0.013	0.040	48.349	45	0.339
Chronic bronchitis		order *Gastranaerophilales*	6	1.061	1.53E-02	−0.007	0.046	0.893	1.313	5	0.934
Chronic bronchitis		phylum *Cyanobacteria*	6	1.055	1.63E-02	0.035	0.042	0.455	3.121	5	0.681
Chronic bronchitis		class *Melainabacteria*	6	1.062	1.39E-02	−0.009	0.046	0.853	1.229	5	0.942
Chronic bronchitis		phylum *Verrucomicrobia*	6	0.965	3.14E-02	−0.008	0.031	0.809	1.388	5	0.926
ILD		genus *Enterorhabdus*	11	0.948	8.33E-03	−0.007	0.009	0.471	7.875	10	0.641
ILD		genus *Peptococcus*	11	0.949	2.45E-02	0.007	0.010	0.490	8.689	10	0.562
ILD		genus *Ruminococcaceae*	11	0.969	2.33E-02	−0.004	0.006	0.498	6.556	10	0.767

### Asthma

The summary GWAS statistics for asthma contain 9,851,867 loci of variants from 21,392 cases and 210,122 controls, according to a definition of asthma. Thirteen causal associations between intestinal taxa and asthma were identified by the IVW method in the sets of IVs (*p* < 5 × 10^−6^). The false discovery rate (FDR) method was used to determine the multiple testing significance at each feature level, and after a rigorous Benjamin and Hochberg correction, a marginal significant causal association between gut microbiota and asthma was identified. In particular, only the genus *Prevotella* [IVW: OR 1.14, 95% confidence interval (CI): 1.062–1.227, *p* = 0.041] was related to a higher risk of asthma. To avoid excessive bias effects, Cochrane’s Q test was performed to analyze the sensitivity of MR results, and no evidence of heterogeneity was found (*p* = 0.688). Moreover, no horizontal pleiotropy was identified by the MR-Egger intercept test (*p* = 0.216) and the MR-PRESSO global test (*p* = 0.657). The leave-one-out test did not find any horizontal pleiotropy in the genus *Prevotella*, and no outlier was found in the genus *Prevotella* by the MR-PRESSO outlier test. In the reverse MR analyses, family Acidaminococcaceae [IVW: OR 0.942, 95% confidence interval (CI): 0.888–0.999, *p* = 0.049], family Enterobacteriaceae (IVW: OR 1.083, 95% CI: 1.024–1.146, *p* = 0.005), order Bacillales (IVW: OR 1.271, 95% CI: 1.065–1.517, *p* = 0.008), order Enterobacteriales (IVW: OR 1.083, 95% CI: 1.024–1.146, *p* = 0.006), genus *Allisonella* (IVW: OR 0.849, 95% CI: 0.729–0.899, *p* = 0.036), genus *Escherichia–Shigella* (IVW: OR 1.065, 95% CI: 1.003–1.131, *p* = 0.041), genus *Phascolarctobacterium* (IVW: OR 0.936, 95% CI: 0.879–0.997, *p* = 0.039), genus *Anaerofilum* (IVW: OR 1.128, 95% CI: 1.027–1.238, *p* = 0.012), genus *Enterorhabdus* (IVW: OR 0.922, 95% CI: 0.855–0.994, *p* = 0.034), genus *Lachnospiraceae* (IVW: OR 1.069, 95% CI: 1.012–1.131, *p* = 0.018), genus *Marvinbryantia* (IVW: OR 0.89, 95% CI: 0.839–0.946, *p* = 1.5e-03), and genus *Peptococcus* (IVW: OR 0.914, 95% CI: 0.838–0.997, *p* = 0.044) have been identified to be significantly linked with an elevated or reduced risk of asthma.

### COPD

A total of 467,437 individuals (49,647 cases and 417,790 healthy controls) were involved in the causal analysis of gut microflora and COPD (22), including the GWAS data from the international COPD Genetics Consortium (ICGC) and FinnGen with additional studies from the UK Biobank. After the MR analysis, 16 bacterial taxa were identified to be associated with COPD, but only the genus *Holdemanella* (OR: 0.850, 95% CI: 0.782–0.924, *p* = 0.0154) presented a tendency to causally decrease the risk of COPD after the correction. The results of other MR analyses were consistent with their respective IVW results. No heterogeneity was observed by Cochrane’s Q test, and the MR-Egger intercept test and MR-PRESSO test also suggested that no horizontal pleiotropy existed. Importantly, the leave-one-out analysis did not detect any outliers in the genus *Holdemanella*. In the reverse analysis, family Streptococcaceae (IVW: OR: 0.946, 95% CI: 0.904–0.924, *p* = 0.019), genus *Defluviitaleaceae* (IVW: OR: 1.068, 95% CI: 1.001–1.141, *p* = 0.048), genus *Peptococcus* (IVW: OR: 1.068, 95% CI: 1.001–1.141, p = 0.048), genus *Ruminococcus* (IVW: OR: 0.934, 95% CI: 0.881–0.989, *p* = 0.022), genus *Erysipelotrichaceae* (IVW: OR: 0.833 95% CI: 0.699–0.991, *p* = 0.039), genus *Streptococcus* (IVW: OR: 0.934, 95% CI: 0.891–0.978, *p* = 0.004), genus *Veillonella* (IVW: OR: 0.931, 95% CI: 0.874–0.991, *p* = 0.026), and genus *Allisonella* (IVW: OR: 1.169, 95% CI: 1.037–1.317, *p* = 0.01) have been found to have a causal relationship with COPD.

### Chronic bronchitis

Next, we focused on dissecting the relationship between chronic bronchitis and intestinal flora, and GWAS data were downloaded from FinnGen (www. https://r9.finngen.fi/). In the sets of IVs (*p* < 5 × 10^−6^), five causal associations from bacterial taxa to chronic bronchitis were identified by the IVW method. After the Benjamin and Hochberg correction, only two bacterial taxa remained stable. Specifically, family Defluviitaleaceae (OR: 3.086, 95% CI: 1.773–5.374, *p* = 0.002) and its child taxon and genus *Defluviitaleaceae* (OR: 3.264, 95% CI: 1.755–6.071, *p* = 0.022) are identified to have a suggestive positive causal effect on the risk of chronic bronchitis. In the sensitivity analysis, no evidence of heterogeneity in the family Defluviitaleaceae (*p* = 0.475) and genus *Defluviitaleaceae* (*p* = 0.298) was observed by Cochrane’s Q test. The results of the MR-Egger intercept test and MR-PRESSO test suggested that no horizontal pleiotropy was found in the family Defluviitaleaceae and its child taxon. The leave-one-out analysis did not detect any outliers in the family Defluviitaleaceae and its child taxon. In the reverse analysis, increasing abundance of the order Gastranaerophilales (IVW: OR: 1.169, 95% CI: 1.037–1.317, *p* = 0.01), phylum Cyanobacteria (IVW: OR: 1.055, 95% CI: 1.01–1.101, *p* = 0.0163), and class Melainabacteria (IVW: OR: 1.062, 95% CI: 1.012–1.114, *p* = 0.014) contributed to the development of chronic bronchitis. In contrast, the abundance of the phylum Verrucomicrobia (IVW: OR: 0.965, 95% CI: 0.934–0.997, *p* = 0.0314) showed a reduced risk with chronic bronchitis.

### ILD

Concerning ILD, GWAS data were downloaded from FinnGen (www. https://r9.finngen.fi/). Twelve causal relationships between bacterial taxa and ILD were observed by the IVW method. After the correction and the cross-validation, only one bacterial taxon remained stable. Specifically, a higher abundance of the genus *Prevotella* (OR 1.347, 95% CI: 1.153–1. 573, *p* = 0.020) was related to a higher risk of ILD. Cochrane’s Q test was used to test the sensitivity of the MR results, and no heterogeneity was identified (*p* = 0.087). Moreover, the MR-Egger intercept test (*p* = 0.136) and MR-PRESSO global test (*p* = 0.111) suggest that no horizontal pleiotropy exists. Importantly, no outliers were identified by the leave-one-out analysis. In the reverse analysis, the abundance of the genus *Enterorhabdus* (IVW: OR: 0.948, 95% CI: 0.912–0.997, *p* = 0.987), genus *Peptococcus* (IVW: OR: 0.949, 95% CI: 0.907–0.993, *p* = 0.024), and genus *Ruminococcaceae* (IVW: OR: 0.969, 95% CI: 0.943–0.996, *p* = 0.023) presented to have a decreased risk with ILD.

### LRTI

As for LRTI, 14,135 cases with 472,349 controls were identified in UK Biobank (23). Ten causal associations from intestinal taxa to LRTI were identified by the IVW method in the sets of IVs (*p* < 5 × 10^−6^). After the Benjamin and Hochberg correction, the family Coriobacteriaceae was identified to have suggestive positive causal effects on the risk of LRTI (IVW OR 1.289, 95% CI: 1.122–1.481, *p* = 0.011). The consistent direction and magnitude of the estimates from other MR analyses further confirmed the causal inferences. Similarly, Cochrane’s Q test indicated that no heterogeneity was found. Moreover, the MR-Egger intercept test (*p* = 0.136) and the MR-PRESSO global test (*p* = 0.111) were used to avoid horizontal pleiotropy. Finally, leave-one-out analysis further supports that the causalities are not driven by any single SNP. In the context of the causal effects of LRTI on gut microbiota, no bacterial taxon was identified to have a causal association with LRTI.

### PAH

PAH is a progressive and incurable vascular disorder characterized by abnormally high blood pressure in the pulmonary artery, contributing to right heart failure with high mortality (24). The genus *Lactobacillus* (OR 5.594, 95% CI: 2.643–14.058, *p* = 0.0297) has been identified to be associated with an increased risk of PAH progression in the set of IVs (*p* < 5 × 10^−6^). The sensitivity of the MR results was assessed by Cochrane’s Q test, and no heterogeneity was identified (*p* = 0.647). Moreover, the MR-Egger intercept test (*p* = 0.603) and the MR-PRESSO global test (*p* = 0.243) suggest that no horizontal pleiotropy exists. Importantly, no outliers were identified by the leave-one-out analysis. In the reverse analysis, no bacterial taxa were suggested to be associated with PAH.

### Lung function

Lung function tests are physiological and non-invasive tests to measure the respiratory function of patients in different situations. In the sets of IVs (*p* < 5 × 10^−6^), the aggregate estimate from all SNPs supported a causal impact of gut microbiota on lung function, especially for FEV1. After the Benjamin and Hochberg correction, the order Selenomonadales was identified to have suggestive negative causal effects on the FEV1(IVW OR 0.931, 95% CI: 0.896–0.968, *p* = 0.011). In the reverse analysis, the IVW analysis did not detect any significant causal associations.

### Ethics statement

The GWAS datasets used in this study were all publicly available. No additional ethical approval was required.

## Discussion

By the use of large-scale GWAS statistics from the UK Biobank and FinnGen, the potential causal relationship between genetically proxied intestinal flora and chronic lung diseases was explored, and five bacterial traits associated with asthma, chronic bronchitis, ILD, LRTI, PAH, and lung function were identified with the framework of TSMR.

Our study suggested that genetic liability to asthma is related to the increased abundance of the genus *Prevotella* among the *Bacteroidetes,* which is a gram-negative bacterium. *Prevotella* is recognized as a member of the oral, vaginal, and gut microbiota and predominates in aspiration pneumonia and pulmonary empyema. In accordance with previous studies, an increased abundance of *Prevotella* at mucosal sites is associated with chronic inflammatory diseases, such as rheumatic diseases and neurodegenerative disorders (25). Santiago et al. have demonstrated that *Prevotella* is one of the most abundant genera among patients with exacerbation-prone severe asthma using deep sequencing of the amplified 16S rRNA gene (26). Moreover, an increasing body of evidence highlights the role of *Prevotella* in modulating the host immune system by impacting the immune compartment within the intestinal tract (27). Specifically, *Prevotella* triggers the release of interleukin-1β (IL-1β), IL-6, and IL-23 from dendritic cells (DC), which, in turn, facilitate the production of IL-17 by T-helper 17 (Th17) cells, and these Th17 cells activate neutrophils and also influence the generation of regulatory T cells (Treg) (25). Considering that IL-17 exhibits pro-inflammatory properties and has been implicated in autoimmunity, its expression could signify a pro-inflammatory function (28). Simultaneously, Treg cells are essential in preventing inflammatory diseases and maintaining immune homeostasis (29). These cytokines govern crucial processes in inflammation and immune response, leading to airway inflammation and bronchoconstriction. Imbalances in the immune system associated with these processes may contribute to the development and exacerbation of asthma. Lopes et al. have shown that the abundance of *Prevotella* in the subgingival biofilm is associated with the presence of severe asthma using quantitative real-time PCR (30). Conversely, Hilty et al. have found that *Prevotella* spp. is more frequent in healthy controls than adult or child asthmatics in the bronchoalveolar lavage fluid (BALF) using 16sRNA sequencing (31), suggesting that the microenvironment of asthma may not be suitable for the colonization, but only 11 patients with asthma are enrolled in this study. However, to date, the role of *Prevotella* in the gut microbiota on asthma remains unknown in the preclinical or clinical studies; we observed the detrimental effects of the bacteria on asthma using the summary-level data, but further experimental and observational studies are needed to dissect the molecular mechanisms of *Prevotella* on asthma. In a similar vein, reverse MR analyses have identified associations between the family Enterobacteriaceae, the order Bacillales, the order Enterobacteriales, and the genus *Escherichia-Shigella* with asthma. A substantial body of research has shown that these four bacterial groups can potentially induce inflammation and infection by producing endotoxins and pathogenic factors (32, 33). For individuals with asthma, an increase in the abundance of these bacteria may heighten their susceptibility to allergic reactions and inflammation. Conversely, the family Acidaminococcaceae, known for its ability to ferment amino acids, has shown potential therapeutic values in asthma (34). The genera *Allisonella* and *Phascolarctobacterium* produce butyric acid and propionic acid, respectively, and an animal study demonstrated that the levels of both were significantly downregulated in asthmatic mice (35). The genus *Marvinbryantia* is capable of fermenting a wide range of carbohydrates, and the protective effects observed can be attributed to the by-products of carbohydrate fermentation (36), which may contribute to the maintenance of intestinal homeostasis and overall immune health. However, less information is available on the genera *Enterorhabdus*, *Anaerofilum*, *Peptococcus*, and *Lachnospiraceae*, and further research is needed to understand their association with asthma.

COPD is a multidimensional chronic lung disease with progressive obstructive bronchiolitis and airflow obstruction (37). In this study, we showed that the genus *Holdemanella* had suggestive negative causal effects on the risk of COPD. Lai et al. have found that *Parabacteroides goldsteinii* and *P. goldsteinii* are able to ameliorate the severity of COPD in a murine cigarette smoking (CS)-induced model (38). Chiu et al. have suggested that the abundance of *Firmicutes* increased in the declining lung function group (39). Chronic bronchitis is included in the umbrella term COPD, defined as productive cough of more than 3 months occurring within 2 years (40). Zheng et al. have suggested that an increase in the total aerobic, *Clostridium perfringens*, *Enterobacter,* and *Enterococcus* significantly increased on the 20th day in a specific pathogen-free Sprague–Dawley rat model with chronic bronchitis (41). Most previous research studies focused on the role of gut microflora on COPD, and few studies have been conducted to investigate the causality of chronic bronchitis on gut microbiota. We found that genetic liability to chronic bronchitis was related to the abundance of the family Defluviitaleaceae, and the genus *Defluviitaleaceae* had a positive correlation with chronic bronchitis; the family *Defluviitaleaceae* belongs to the order *Clostridiales* which is associated with worse recurrence-free survival (RFS) in patients with non-small cell lung cancer (42). The genus *Defluviitaleaceae* belongs to the family *Lachnospiraceae*, with a sequence similarity of the 16S rRNA gene of approximately 87%. In this study, we also have performed the MR analysis to explore the causality between COPD and gut microbiota and found that the genus *Holdemanella* presented a tendency to causally decrease the risk of COPD after the correction. However, Bowerman et al. have suggested that some bacterial taxa, including *Streptococcus sp000187445*, *Streptococcus vestibularis,* and several members of the family *Lachnospiraceae* correlated with reduced lung function and COPD (22). Similarly, Jang et al. have shown that the increased *Defluviitaleaceae* was found in the gut microbiota of emphysema compared with the healthy controls using pyrosequencing (43).

ILDs are a heterogeneous spectrum of disorders that principally influence the pulmonary interstitium, resulting in dyspnoea, cough, and respiratory failure (44). Chioma et al. have demonstrated that gut microbiota regulates lung fibrosis severity followed by acute lung injury (45). Using GWAS data, we found that the abundance of the genus *Prevotella* has a positive correlation with ILDs. Huang et al. have suggested that the activation of immune response signaling pathways is strongly related to the reduced abundance of *Prevotella* among individuals with fibroblasts responsive to CpG-ODN stimulation (46). Scher and Lou have identified that *Prevotella* in the lungs is associated with the initiation and development of ILD in patients with autoimmune diseases, such as dermatomyositis and rheumatoid arthritis (RA) (47, 48). To date, no relevant data have reported on the gut microbiome of ILD or IPF in humans, but Gong et al. have found that the abundance of *Alloprevotella, Dubosiella, Helicobacter, Olsenella, Parasutterella, Rikenella*, and *Rikenellaceae RC9 gut group* in the gut of the bleomycin (BLM) or silica-induced mice present significant difference compared with the healthy controls by 16S RNA sequencing (49).

The influence of *Prevotella* on ILD encompasses not only the immunological factors, as previously discussed, but also microbial interactions. The gut-lung axis embodies the idea that changes in gut commensal microorganisms can exert distant effects on immune function in the lung (50) while simultaneously involving gastrointestinal functionality and intricate bidirectional communication with the respiratory system (51). Under certain conditions, such as systemic circulation or inhalation of gastroesophageal reflux, *Prevotella* may translocate to the lung tissue via the enteropulmonary axis. The presence of enteric bacteria in the lungs can initiate a local immune response, leading to inflammation and tissue damage, which may subsequently contribute to the development of ILD46. *Prevotella* generates various metabolites and signaling molecules, such as SCFA47. These molecules traverse the gut-lung axis, affect local immune responses, and could potentially induce inflammation and fibrosis in the context of ILD.

LRTI is an umbrella terminology, including acute bronchitis, pneumonia, acute exacerbation of COPD (AECOPD), and acute exacerbation of bronchiectasis (52), which is the most common cause of death in low-income countries (53). In this study, we found that the abundance of the family Coriobacteriaceae and class Coriobacteriia affects the occurrence of LRTI. It is known that interstitial flora contributes to LRTI pathogenesis and severity through its immunomodulatory properties (54, 55). Goossens et al. have shown that the abundance of the family Coriobacteriaceae tended to be elevated in the gut after intraperitoneal LPS challenge, which is recognized to be associated with the increased expression of matrix metalloproteinases (MMP9) (56). However, Sencio et al. have observed an obvious reduction of Actinobacteria (Bifidobacteriaceae and Coriobacteriaceae families) in the cecal samples from influenza A virus-infected patients (57), and the alteration of SCFAs by the interstitial flora influences the killing activity of alveolar macrophages (57).

PAH is a malignant and devastating pulmonary vascular disorder characterized by precapillary pulmonary hypertension. In this study, we found that the genus *Lactobacillus* had a causal role in PAH. Mounting evidence suggests that the gut-dwelling *Lactobacillus* and its components play a key role in modulating the immune system through stimulating immunological signaling between the gastrointestinal tract and distant organs (58). Consistently, Ma et al. have also demonstrated that the increased abundance of *Lactobacillus* is associated with PAH compared with healthy volunteers and congenital left to right shunt heart diseases (59).

Lung function is used to measure lung volume, capacity, and flow rates, reflecting the functional status of the lungs and the disease severity. We found that the order Selenomonadales pertaining to *Veillonella* presents a reduced risk with FEV1, which is useful to categorize the severity of obstructive lung diseases such as COPD. In contrast, Diao et al. have shown that the abundance of the order Selenomonadales was significantly increased in the throat microbial flora in COPD (60). Moreover, Filho et al. have demonstrated the absence of the order Selenomonadales in the adult lungs, which were independent predictors of mortality in COPD. However, to date, no available studies have been conducted to investigate the role of the order Selenomonadales in the gut on obstructive lung diseases.

It is well-established that microorganisms not only can be found in the gut but also in the respiratory tract (8). In the upper respiratory tract, there are variations in the microbial composition based on the location. For instance, the nasal cavity and nasopharynx are primarily populated by *Moraxella*, *Staphylococcus*, *Corynebacterium*, *Haemophilus*, and *Streptococcus* species, while the oropharynx contains a high abundance of *Prevotella*, *Veillonella*, *Streptococcus*, *Leptotrichia*, *Rothia*, *Neisseria*, and *Haemophilus* species (61). On the other hand, the lower respiratory tract, which includes the trachea and lungs, maintains a relatively low microbial biomass, which is crucial for lower airway mucosal immunology, as it allows for swift microbial clearance through various physiological mechanisms. Extensive research is currently underway to understand how the gut microbiota impacts immune responses and inflammation in the lungs, and conversely, how the lungs influence the abundance of gut microbiota. Various mechanisms, such as the participation of specific subsets of regulatory T cells (62, 63), Toll-like receptors (TLRs), inflammatory cytokines, mediators, and numerous other factors, have been suggested as potential explanations for these interactions (64). However, the precise biological mechanisms remain largely unknown.

The strength of this study is that we employed bidirectional and comprehensive MR that exploits genetic variants to estimate the causal effects of gut microbiota on chronic lung diseases, and MR is capable of minimizing bias due to confounding and reverse causality, thus improving the causal inference (65). Moreover, we performed the analysis with large sample sizes which promotes the power to detect mild-to-moderate associations, and individuals included in this study are all European ancestry to reduce the population selection bias. Nevertheless, this study had several limitations. First, the number of IVs involved in GWAS statistics of gut microbiota is small, and no additional data are available at the species level, which contributes to biased estimates and lack of universality. Second, the methods of sequencing analyses of the gut microbiota and chronic lung diseases may differ, leading to distinct results. Third, the phenotypes of the six lung diseases were not analyzed in this study. Finally, due to the summary-level GWAS data, the demographic data of the studies are absent; further subgroup analysis of the confounding factors such as age and gender on the bacterial taxa and lung diseases remains unknown.

## Conclusion

Our bidirectional TSMR study reveals the causal relationship between gut microbiota and chronic lung diseases, providing new insights into the biological mechanisms of gut microbiota-modulated development of chronic lung diseases. To facilitate the dissection of the role of gut microbiota on lung diseases, an integrative approach that uses multiple omics is urgently needed to understand gut-lung signaling.

## Data availability statement

The datasets presented in this study can be found in online repositories. The names of the repository/repositories and accession number(s) can be found in the article/Supplementary material.

## Ethics statement

Ethical approval was not required for the study involving humans in accordance with the local legislation and institutional requirements. Written informed consent to participate in this study was not required from the participants or the participants’ legal guardians/next of kin in accordance with the national legislation and the institutional requirements.

## Author contributions

YS: Writing – original draft, Writing – review & editing. YZ: Writing – original draft, Writing – review & editing. JX: Supervision, Writing – original draft, Writing – review & editing.
